# What is new in burn care?

**DOI:** 10.4103/0970-0358.70711

**Published:** 2010-09

**Authors:** Surajit Bhattacharya

**Affiliations:** Editor, IJPS, Sr. Consultant Plastic Surgery, Sahara Hospital, Lucknow, India. E-mail: surajitb@sancharnet.in

A burn injury is not limited to the skin but is associated with the dysfunction of almost every cell in the body and various organs leading to alterations, not only in cellular functions but also in cellular signaling pathways. The current thought that alterations and new treatments of these pathways may restore cellular function and productivity, which in turn may lead to an improved energy metabolism, cell survival, morbidity, and mortality is perhaps the most cutting-edge research in burn care. Hypermetabolism is associated with cell deaths (apoptosis) in skeletal muscles, fat, liver, kidney, lung, heart and gut. These detrimental cellular events are also associated with dysfunction of the cellular organelles such as the mitochondria or the endoplasmic reticulum, thus making burn a truly systemic disease.

The areas of advancement in burn care that have improved results of both acute injuries as well as long-term outcome include acute care, burn wound treatment, control of the hypermetabolic responses, and control of life-threatening infections. Both the mortality as well as the length of hospital stay of burned patients have been greatly reduced because of improvement in acute care, such as resuscitation and management of inhalation injuries, early excision and grafting, the control of infections and improving the immune system, improvements in provision of metabolic and nutritional requirements, evolution of effective skin banks, infection control, and alternative wound-closure materials and strategies.

Gene therapy is another emerging technique to alter dermal and epidermal regeneration and to improve wound healing. Non-viral constructs have been shown to effectively change the cells into a productive and active cell that releases growth factors and other signaling factors to improve dermal and epidermal regeneration. Liposomal gene constructs have been shown to transfect dermal stem cells increasing the therapeutic possibilities of this futuristic technology!

Skin substitutes are creating a revolution and it is now possible to construct an artificial skin, which is called cultured skin substitute (CSS). CSS is the patients own skin (epithelium) in combination with a dermal substitute (Integra) that represents a full-skin substitute including both dermis as well as epidermis. The use of bilayer artificial skin has improved the survival and cosmetic results of early eschar excision in patients with massive full-thickness burns. This technique is helping both, in the acute stage to cover large raw areas as well as in reducing the amount of donor sites taken from the patient and improving cosmesis associated with an improved quality of life in severely burned patients. The challenge that remains is to make this science economically viable for those who need it most.

The scope of surgery too has changed from covering chronic burn wounds and treating disfiguring deformities to management of the acute burn wound. Thus escharectomy, tangential excision, meshed skin grafting, micro-skin grafting, and meek grafting are all invaluable arsenals of our armamentarium. Softened freeze-dried glutaraldehyde-preserved skin, chlorhexidine-alcohol refrigerated porcine skin, and frozen amniotic membrane are all effective as burn dressings. The reconstruction in the burn patient is often a long process requiring multiple procedures and stages. It demands a stepwise and prioritized approach, aiming at both maximum function as well as optimal appearance. With better anaesthetic and critical care support surgeons do not hesitate to climb up the reconstructive ladder and microsurgical reconstructions for the sake of better function and cosmesis are becoming more and more common.

Rehabilitation of burn patients to effectively reintegrate them in the society as productive members has been a Herculean task, which is still ongoing. Vocational training has rendered these patients the skills and developmental improvements that are truly outstanding, leading to their return as useful and productive members of their family. The devastating effects of burns are long lasting at both an individual and societal level. These impacts are compounded in resource-poor settings, where the human and material resources necessary to deal with this complex public health problem are thinly distributed over a large population base. A structured and comprehensive approach to burn care must be applied to these resource-poor settings in order to improve outcomes.

A combination of improved management and prevention strategies has resulted in important declines in morbidity and mortality in the developed world. Unfortunately, without adequate resources in first-aid, acute surgical management and rehabilitation facilities, patients that do survive their burn injuries in developing countries often have poor, disfiguring and disabling long-term outcomes. Burn care truly deserves a helping hand from both governmental and non-governmental sources and philanthropists should come up with a modestly funded burn care strategy for the developing world.

